# Click to Correction: Interactive Bidirectional Dynamic Propagation Video Object Segmentation Network

**DOI:** 10.3390/s24196405

**Published:** 2024-10-02

**Authors:** Shuting Yang, Xia Yuan, Sihan Luo

**Affiliations:** 1 Institute of Agricultural Economy and Information Technology, Ningxia Academy of Agriculture and Forestry Sciences, Yinchuan 750002, China; nxnkyyst@163.com; 2 School of Computer Science and Engineering, Nanjing University of Science and Technology, Nanjing 210094, China; luosihan@njust.edu.cn

**Keywords:** video object segmentation, interactive segmentation, click-based interactive, bidirectional propagation

## Abstract

High-quality video object segmentation is a challenging visual computing task. Interactive segmentation can improve segmentation results. This paper proposes a multi-round interactive dynamic propagation instance-level video object segmentation network based on click interaction. The network consists of two parts: a user interaction segmentation module and a bidirectional dynamic propagation module. A prior segmentation network was designed in the user interaction segmentation module to better segment objects of different scales that users click on. The dynamic propagation network achieves high-precision video object segmentation through the bidirectional propagation and fusion of segmentation masks obtained from multiple rounds of interaction. Experiments on interactive segmentation datasets and video object segmentation datasets show that our method achieves state-of-the-art segmentation results with fewer click interactions.

## 1. Introduction

Video object segmentation (VOS) aims to segment objects from the background and obtain a pixel-level mask. Complex background, motion blur, view angle change, occlusion, and scaling make high-quality VOS very difficult. In recent years, with the development of deep learning, many end-to-end VOS models have been proposed. However, how to predict a more accurate object mask is still a challenging problem.

**Figure 1:**
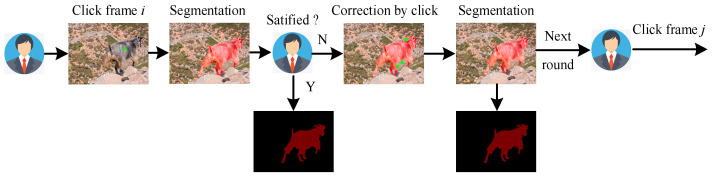
Illustrating of click-based multi-round interactive video object segmentation.

Research has shown that introducing user interaction in target segmentation tasks can significantly improve the quality of generated masks [1]. It allows users to carry out various forms of interaction in a single video frame, such as mouse clicks, box selection, and graffiti, find all pixels belonging to the target object according to the interactive information, and output the interactive segmentation mask. The user can continue to supplement the interaction according to the predicted segmentation result to correct the previous errors through positive and negative interaction until the output result is satisfactory. Figure 1 shows an example of multi-round interactive segmentation.

Clicking is simply and currently the most popular interaction method. Click-based interactive segmentation is one of the widely studied topics [2,3]. F-BRS [4] is a backpropagating refinement scheme that operates on intermediate features in the network and requires running forward and backward passes just for a small part of a network. Experiments demonstrated a better convergence of backpropagating refinement schemes compared to pure feed-forward approaches. FocalClick [5] decomposes the slow prediction on the entire image into two fast inferences on small crops: a coarse segmentation on the Target Crop, and a local refinement on the Focus Crop. It formulates a sub-task termed Interactive Mask Correction and proposes Progressive Merge as the solution. It shows superiority when making corrections on pre-existing masks. Ref. [6] regards interactive segmentation as a pixel-wise binary classification problem and proposes GPCIS, a Gaussian process classification framework. To solve the proposed model, it has been proposed to variationally approximate the GP posterior in a data-driven manner, along with a decoupled sampling strategy with linear complexity. Ref. [1] presents a technique for automatically estimating the quality of the produced masks, which exploits indirect signals from the annotation process. It has shown that interactive segmentation can be a compelling approach for instance segmentation at scale. MiVOS [7] presents a modular interactive VOS framework that decouples interaction-to-mask and mask propagation, allowing for higher generalizability and better performance.

Many traditional researchers have adopted technologies such as a clustering algorithm, graph model, random walk, random decision forest, and Markov random field to deal with VOS. Deep learning-based VOS models have been rapidly developed since 2015, surpassing traditional methods [8]. VOS methods can be divided into the following three categories, which are online optimization-based, matching-based, and propagation-based methods.

Methods based on online optimization [9,10,11,12] focus on optimizing the first frame of the video to accurately segment objects and show an effective performance. However, its computational cost is high in application. When the object undergoes significant deformation, the algorithm performance deteriorates.

Matching-based work [13,14,15,16] usually calculates the pixel-level or feature-level matching score between the template frame and the current prediction frame to segment video objects. The matching score is generally obtained by calculating each pixel in the current prediction frame and the nearest neighbor pixel in the template frame. In scenes with complex backgrounds or fast changes in objects, the performance of matching-based methods will be significantly reduced due to the lack of global guidance.

Propagation-based work [7,17,18,19,20,21,22,23] utilizes previously obtained masks or learned feature embedding to obtain better VOS performance. The main idea is to use the temporal and spatial consistency of the target object in the video, such as optical flow, to assist the mask prediction of the current frame. The propagation-based model has many parameters and the propagation process can lead to the accumulation of errors. The temporo-spatial memory network has been a key research method in the field of video segmentation in recent years [24].

Large-scale, accurately labeled image data are one of the key prerequisites for the success of deep neural networks in video segmentation. However, collecting and labeling these datasets requires significant manpower and resources, which makes video segmentation datasets contain annotation noise. So, learning from noisy labels is an effective method to improve video segmentation quality. For example, ref. [25] proposes BPT-PLR, a balanced partitioning and training framework with pseudo-label relaxed contrastive loss. It introduces a balanced partitioning process with a two-dimensional Gaussian mixture model and a semi-supervised oversampling training process with a pseudo-label relaxed contrastive loss.

This paper combines the advantages of propagation-based and matching-based methods and proposes a multi-round interactive dynamic propagation video object segmentation network (MRIDP_VOS). The network consists of two parts: interaction segmentation and dynamic propagation. The user interaction segmentation module converts the click-based interaction into an instance mask, and the dynamic propagation module propagates it to adjacent frames to predict accurate instance masks in the entire video. Users can dynamically improve segmentation results through multi-round interaction. The contributions of this paper are as follows.

(1) A multi-round interactive learning video object segmentation network MRIDP_VOS is proposed, which consists of a click interaction segmentation module and a bidirectional dynamic propagation segmentation module. In the interactive segmentation module, a priori segmentation backbone combined with a high-resolution feature extraction network and a convolutional block attention module is proposed to extract a deep semantic representation of objects with different scales clicked by users.

(2) A bidirectional temporal mask propagation module is proposed to calculate the differences between masks predicted for different rounds of interaction during mask propagation and correct the current prediction results. It preserves the user’s intention for different rounds of interaction.

(3) We design an optimization module to fuse segmentation masks in different frames of different interaction rounds. This module can retain the user’s intentions in different rounds of interaction.

## 2. Methods

**Figure 2:**
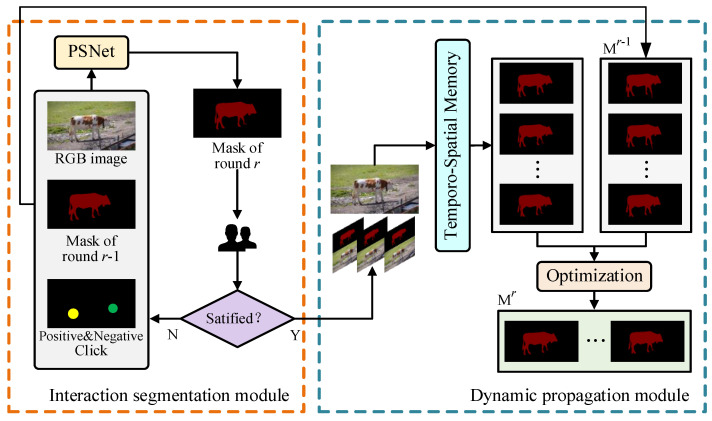
The structure of MRIDP_VOS network.

The framework of the proposed MRIDP_VOS is in Figure 2. It is composed of a user interaction segmentation module and a dynamic propagation module. The user interaction segmentation module receives user clicks to generate a segmentation mask in real time by using a prior segmentation network (PSNet). The dynamic propagation module propagates the mask through the temporo-spatial memory network. And then it uses the optimization module to fuse the mask of the current round with the previous round to generate the final segmentation mask. MRIDP_VOS allows users to interact in multiple rounds until they are satisfied with the segmentation result of the whole video.

### 2.1. User Interaction Segmentation Module

**Figure 3:**
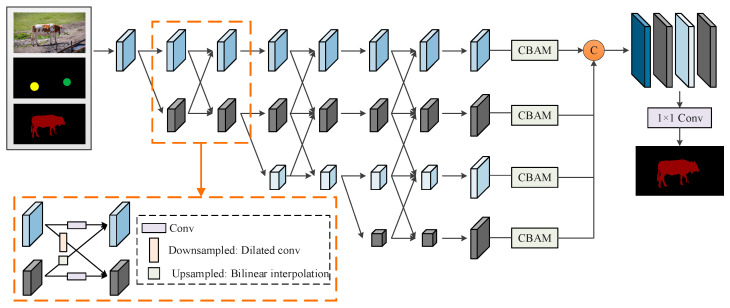
Structure of PSNet in interaction segmentation module.

The user interaction segmentation module realizes the positioning, segmentation, and correction of the foreground mask through a small amount of click interaction. The overall structure of the PSNet network in the user interaction segmentation module is shown in Figure 3.

This paper designs a combination of high-resolution network [26] and CBAM [27] attention module as the backbone of PSNet. The high-resolution network generates high-, medium-, and low-resolution feature maps to fully explore the deep representation of objects of different sizes. CBAM is used to capture the features in different channels after the output of each layer in the high-resolution network. After feature extraction, the full connection layer is used to fuse different scale features.

The inputs of the PSNet include RGB image, click map, and segmentation mask. The click map is a single-channel image created by the user’s selective positive and negative clicks on one frame of the video. The mask is the object predicted in the previous round of interaction. If it is the first interaction, a blank mask is used as input. We use the mask predicted in the previous round to assist in the segmentation of the current frame, thereby associating it with the results of multiple rounds of interactive prediction.

### 2.2. Dynamic Propagation Module

The dynamic propagation module includes two parts: temporo-spatial memory network and an optimization module. Given an object mask, the temporo-spatial memory network tracks the object and generates the corresponding mask in the subsequent frame to propagate the mask bidirectionally in the time domain. The optimization module fuses the transmitted mask and the previous round of masks more accurately and smoothly to avoid forgetting the user’s intention of the previous round of clicks and ensure the temporal and spatial consistency of video segmentation results.

#### 2.2.1. Temporo-Spatial Memory Network

**Figure 4:**
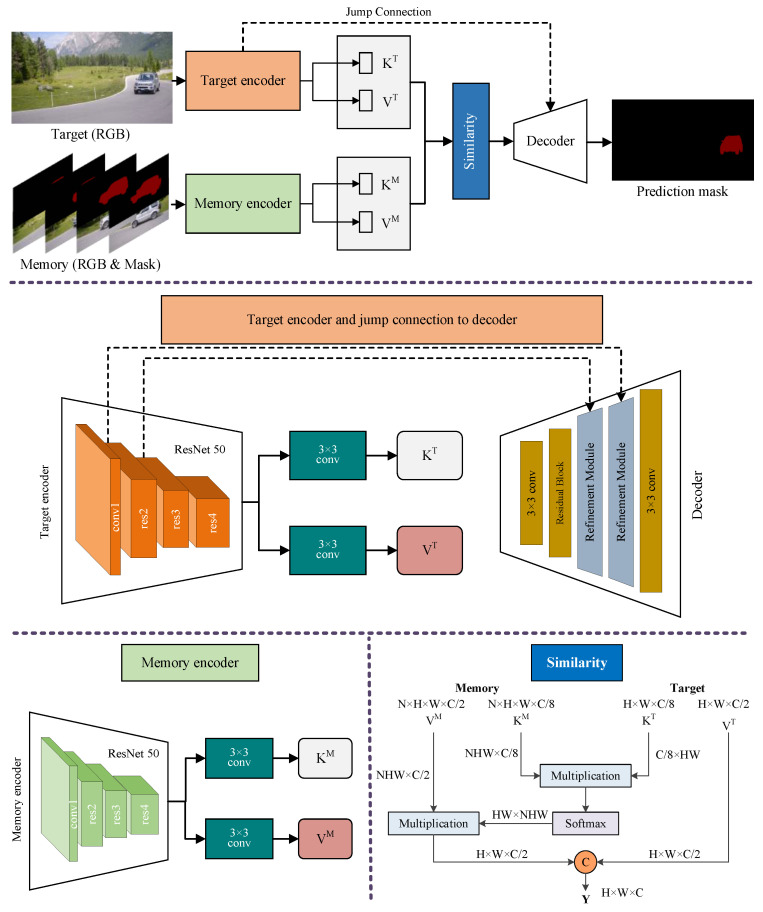
Structure of temporo-spatial memory network.

The structure of the temporo-spatial memory network is shown in Figure 4. The current frame is called target and the past frames are called memory. The memory encoder and target encoder are built to extract object features and implement query operations. The memory encoder encodes the object into key–value pairs, where the key is used for query and the value is used for subsequent attention calculation. The target encoder extracts the features of the current frame for matching and associating with the objects in memory. The input of the memory encoder is RGB images and the corresponding masks stacked along the channel, and the input of the target encoder is an RGB image.

Key and value form a key–value pair. Vector key is used to evaluate the similarity between the target frame and the memory frame, and determine which features in the memory frame should be sampled. The vector value is used to generate the result mask. After key–value pairs of target and memory are obtained, the target frame is associated with the memory frame, that is, to retrieve frames in memory that are like the target frame. This process is called similarity calculation, as shown in the lower right of Figure 4.

The decoding module of the temporo-spatial memory network receives the output of the similarity calculation module and the features of the target encoder. We replaced the convolutional layer with a residual block in the decoder and named the refinement module in Figure 4. The decoder of the temporo-spatial memory network includes two refinement modules with the same structure, two 3 × 3 convolutional layers, and a residual block to generate the target mask, with the output mask being 1/4 of the input image size. Each convolutional layer of the refinement module generates a feature map with 256 channels, and, finally, a 2-channel mask map is generated through a 3 × 3 convolutional layer.

#### 2.2.2. Optimization

1. Mask Bidirectional Propagation

**Figure 5:**
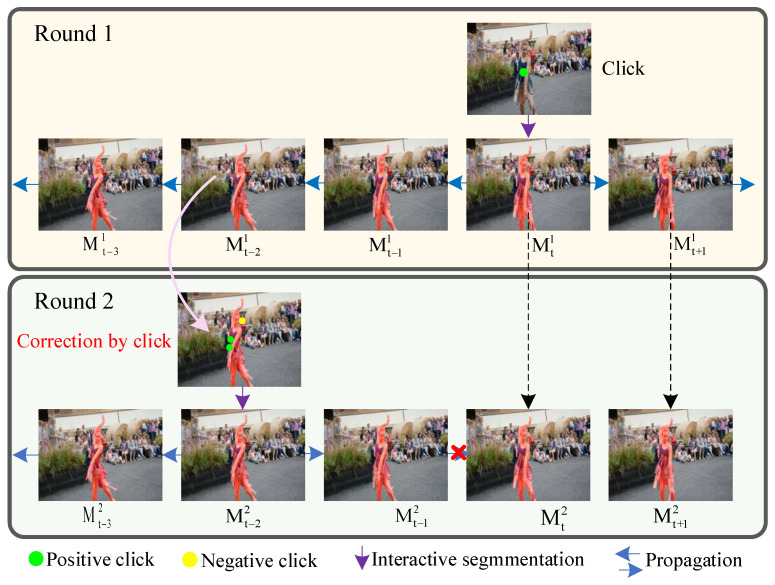
Schematic diagram of mask bidirectional propagation.

The dynamic propagation module propagates and derives the segmentation mask of one frame to other video frames. Considering that mask propagation frame by frame may lead to error accumulation, this paper adopts a bidirectional propagation method similar to [7], as shown in Figure 5. $M_{t}^{r}$ is the mask of frame t after interaction round r. The mask is propagated in the forward and backward directions until the interactive frame is encountered or the start and end of the video are reached. Masks of interactive frames are reliable.

2. Optimization module

Since the mask propagation is independent of the masks predicted in previous interaction rounds, the intention of the user in previous interaction rounds may be lost. In order to avoid this situation, the segmentation mask obtained in the current round needs to be corrected by the mask obtained in the previous round. We use ΔM to present the difference between $M_{t}^{r-1}$ and $M_{t}^{r}$, and capture the user’s intent.

**Figure 6:**
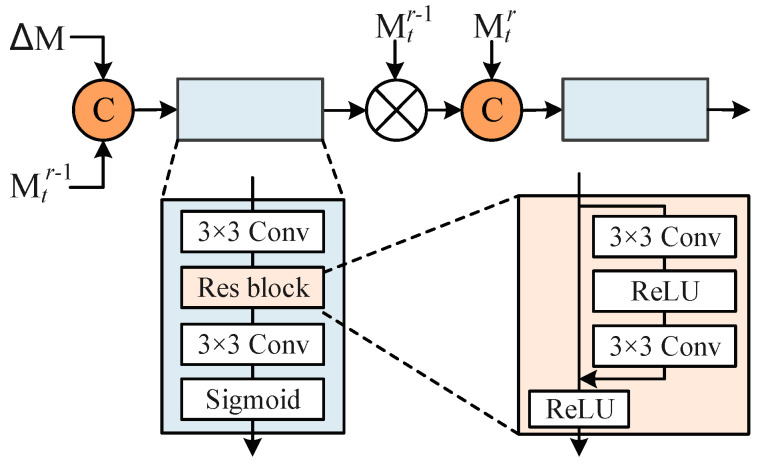
Structure of optimization module.

The structure of we designed optimization module is shown in Figure 6. The input to the module is the feature map cascade by ΔM and $M_{t}^{r-1}$. The optimization module aims to use the difference between the features of the current round and the previous round to calculate the weight of the features of the previous round and use the weight to supplement the feature information of the previous round to the current round. These differences are input into the optimization module as guidance information to help the optimization module better combine the propagated mask with the mask of the previous round to obtain a more accurate prediction mask.

## 3. Experimental Results

### 3.1. Datasets, Metrics, and Implementation

#### 3.1.1. Datasets

The user interaction segmentation module and dynamic propagation module in MRIDP_VOS are trained independently. This paper uses the SBD [28] training set to train the user interaction segmentation model and verifies it on the SBD test set, DAVIS [29], and Berkeley [30] dataset. There is no user interactive click information in these datasets. In order to generate click information similar to multiple rounds of interaction with real users, this paper uses random sampling at the beginning and then employs the iterative sampling program proposed by [31] in the subsequent process to generate all subsequent clicks for the image.

We use DVIS 2017 [32] as the training data of the video segmentation model, without using any additional training data. MRIDP_VOS is validated on the DVIS 2017 validation set.

#### 3.1.2. Metrics

The user interaction segmentation module uses NoC@85 and NoC@90 as evaluation metrics, that is, the number of clicks required to achieve 85% and/or 90% IoU. Regional similarity *J*, contour accuracy *F*, and the average of them Avg(*J*&*F*) are used to evaluate the results of video object segmentation.

Region similarity *J* is constructed based on area, which measures the similarity between the predicted segmentation results and the ground truth from the perspective of the region. The set of annotated masks for all frames of the video is defined as **G**, and the set of predicted masks is defined as **M**. Regional similarity *J* represents the proportion of correctly predicted pixels in the predicted mask to all pixels by calculating the intersection to union ratio, and is calculated by (1).

(1) $$J=\frac{\left|M\cap G\right|}{\left|M\cup G\right|}$$

The contour accuracy *F* evaluates the similarity between the predicted mask **M** and the ground truth **G** from the perspective of contour. *F* is calculated by (2)–(4).

(2) $$F=\frac{2P_{C}R_{C}}{P_{C}+R_{C}}$$

(3) $$P_{C}=\frac{TP}{TP+FP}$$

(4) $$R_{C}=\frac{TP}{TP+FN}$$

where *TP* (true positive) represents the number of boundary pixels predicted as objects and belonging to ground truth boundary pixels at the same time; *FP* (false positive) refers to the number of boundary pixels predicted as objects but not belonging to ground truth boundary pixels; *FN* (false negative) refers to the number of boundary pixels that are not predicted as objects but belong to ground truth boundary pixels.

#### 3.1.3. Implementation

Experiments in this paper were run on the Ubuntu 18.04 operating system. MRIDP_VOS was trained by four NVIDIA TITAN XP GPUs, and inferenced by a single GPU. The experimental code was written by Python 3.8 and PyTorch 1.6.0. The user interaction segmentation module used Adam optimizer with a momentum of 0.9. The cross-entropy function was used to calculate the loss. The number of training batch sizes was set to 8 and the model training process included 55 epochs. The initial learning rate was set to 5 × 10^−5^ and was reduced to 5 × 10^−6^ after the 50 epochs.

The dynamic propagation module used an Adam optimizer with batch size 8, the learning rate was set to 5 × 10^−5^, and the training included 60 epochs. In each epoch, three consecutive frames were randomly selected from the video sequence to form a training batch. The first frame was used as the ground true mask frame, and the first frame was used to predict the result of the second frame. Then, the predicted second frame was used as the new reference frame, compared with the first and third frames to calculate their similarity and predict the mask of the third frame.

### 3.2. Evaluation of User Interaction Segmentation

The proposed user interaction segmentation module was compared with seven typical interactive-based segmentation models on the SBD, Davis, and Berkeley datasets.

**Figure 7:**
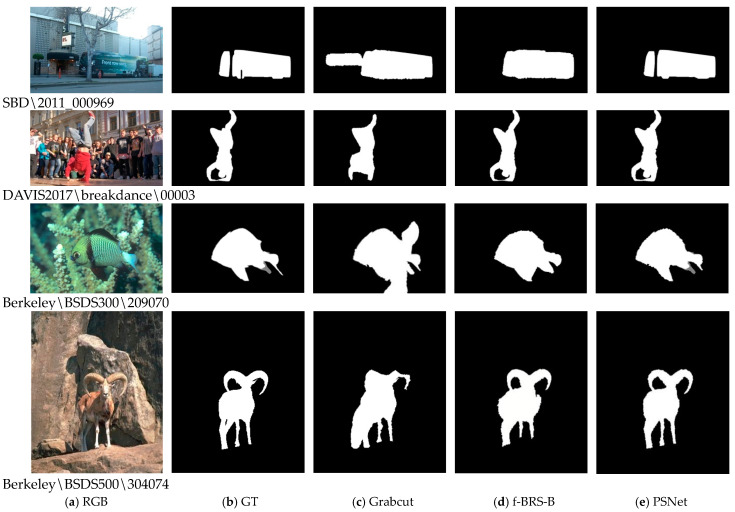
Results of qualitative comparison.

In Table 1, the proposed PSNet achieved the best performance on the Davis and Berkeley datasets and the second-best on the SBD dataset. Figure 7 shows the comparison of the segmentation results of Grabcut, f-BRS-B, and PSNet. Grabcut has a significantly worse segmentation result than modern methods. f-BRS-B is significantly improved compared to Grabcut, but not as good as PSNet. For example, for the bus and gazelle in Figure 7, the PSNet can segment the edge smoothly and accurately, and some occluded areas can also be well separated.

PSNet uses a high-resolution network combined with CBAM to extract different scales of features through multiple branches at the same time and integrates these features to extract user interactive information more accurately. The quantitative and qualitative experimental results indicate that the proposed PSNet can achieve better interactive segmentation results.

### 3.3. Evaluation of Video Object Segmentation

#### 3.3.1. Comparison to the State of the Art

Eight video object segmentation methods are selected for comparison with MRIDP_VOS, which are IPN, ATNet, GNNannot, MANet, MiVOS, XMem, ISVOS, and MED-VT. Methods used for comparison are compared using the results obtained from training and validating on the DAVIS 2017 dataset, without using any additional training data.

Table 2 shows quantitative results on the DAVIS 2017 (instance level VOS dataset) validation set. MRIDP_VOS obtains the best result among six interactive segmentation methods and the second best of *J* and *F* of all compared methods. MED-VT is an object-level segmentation method, so the score reported in [23] is on DAVIS 2016 (object-level VOS dataset). Because instance segmentation is more challenging than object-level segmentation, the score on DAVIS 2017 is lower than that on DAVIS 2016. Based on the comprehensive analysis of the experimental results in Table 1, our method is highly competitive compared to the state of the art. It is worth noting that interactive methods are based on interactive information for segmentation, without using the mask annotated in the first frame of the video in the DAVIS dataset, making segmentation more difficult. Therefore, the segmentation results based on interactive methods are often slightly inferior to the semi-supervised video object segmentation method that uses the ground truth mask in the first frame.

MiVOS has been a representative interactive VOS method in recent years. From Table 2, MRIDP_VOS outperforms MiVOS, and the click interaction used by MRIDP_VOS is more efficient and user-friendly than the graffiti interaction used by MiVOS.

**Figure 8:**
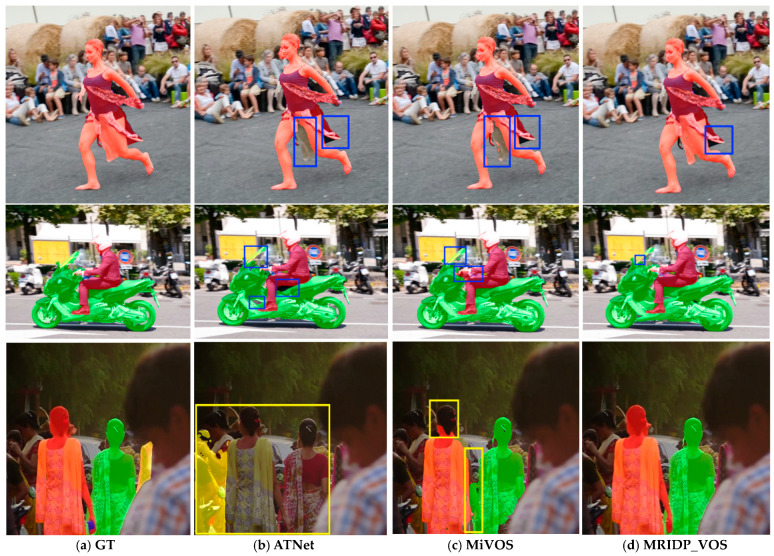
Results of qualitative comparison. The bounding boxes highlight the difference between different methods.

Figure 8 shows a comparison of segmentation results. Both ATNet and MiVOS did not fully segment the skirt of the dancer in the first row, while the MRIDP-VOS model was able to correctly segment the middle part of the skirt. In the video of the second pedestrian riding a motorcycle, ATNet has many under-segmented areas. MiVOS mistakenly segments the motorcycle handle as a part of the human hand. MRIDP_VOS separates both people and motorcycles relatively completely, with only smaller areas not being correctly segmented. In the third row, ATNet failed to segment two people, and MiVOS did not segment the head and arm of the left people well. The result of MRIDP_VOS in the third row is the closest to the ground truth. The experimental results in Figure 8 indicate that PSNet has stronger feature extraction capabilities than the DeepLabv3+ used in MiVOS, resulting in better segmentation results after multi-round click interaction.

**Figure 9:**
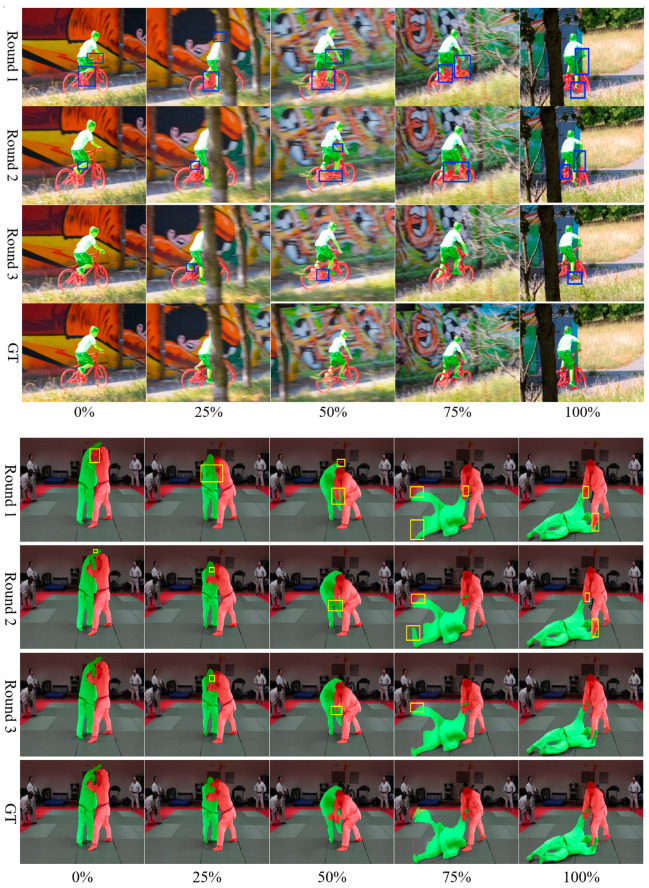
A 3-round interactive video segmentation results of MRIDP_VOS. The bounding boxes highlight the difference between different methods.

Figure 9 shows the three-round interactive segmentation effect of MRIDP-VOS on a segment of two video clips in the DAVIS 2017 validation set, and 0%–100% represents the progress of the video. As the number of interaction rounds increases, the segmentation results become more accurate. Under the guidance of the first round of click interaction, there are obvious mis-segmentation and under-segmentation areas between human and bicycle, and two judo practitioners. However, after the second and third rounds of interaction, the mis-segmentation and under-segmentation areas gradually decrease, and the boundaries between people and objects, as well as between objects and backgrounds, are becoming increasingly accurate.

#### 3.3.2. Ablation Study

We designed three ablation experiments to demonstrate the effectiveness of each module of MRIDP-VO:

(i) Replace the backbone network in PSNet with DeepLabv3+;

(ii) Remove interactive information in the dynamic propagation module and only propagate the preceding frames and masks;

(iii) Remove the optimization module from the dynamic propagation module.

Table 3 presents a quantitative comparison of the segmentation performance between the model variants generated by these three ablation experiments and the MRIDP-VOS standard model. After replacing the backbone network of PSNet with DeepLabv3+, the Avg(*J*&*F*) decreased the most, from 82.4% to 77.0%, especially contour accuracy F, which decreased from 84.9% to 77.3%. This indicates that PSNet has played an important role in improving the details of the object contour. After removing the interaction information in the dynamic propagation module, Avg(*J*&*F*) decreased by 5.2%, indicating that timely user clicks to correct during the propagation process play an important role in predicting other frame masks. After removing the optimization module, Avg(*J*&*F*) decreased by 6.1%, indicating that the optimization module can effectively integrate the current round of masks with the previous round of masks, eliminate conflicts, effectively capture user intentions, and more accurately segment video objects.

**Figure 10:**
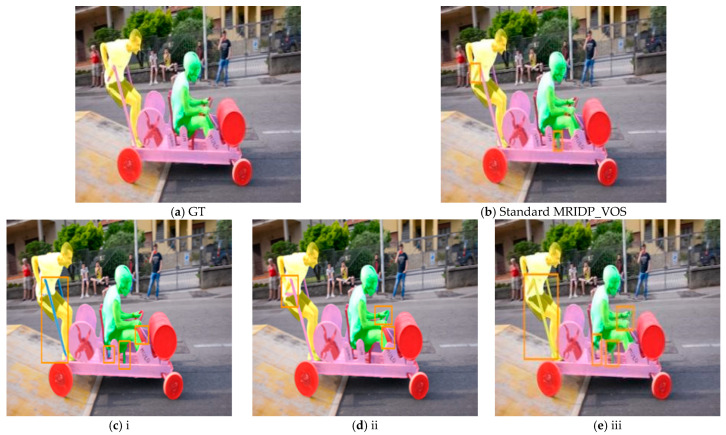
Qualitative results of ablation study.

Figure 10 shows a qualitative comparison of segmentation results between various ablation experimental model variants and the MRIDP-VOS standard model. Compared to the other three variant experiments, the MRIDP-VOS standard model has the smallest error segmentation area in the results. The use of DeepLabv3+ as the backbone network, removal of interactive information, and removal of optimization modules significantly lead to poorer segmentation results and more erroneous segmentation areas in the model.

#### 3.3.3. User Interaction Study

The purpose of interactive video segmentation is to obtain high-precision segmentation results. Real-time performance is not the first consideration for this task. To evaluate the effectiveness and efficiency of our method during the execution process, we conduct a user study to evaluate the human effort required to interactively segment a video using the proposed method. Specifically, we quantify the required human effort by the total user time, which includes the time for interaction, searching, or pausing to think, while excluding all computational time. We compare this with MiVOS [7], which is a good-performing method with available source code.

We recruited five volunteers who were given sufficient time to familiarize themselves with MRIDP_VOS and MiVOS and the GUI. They were asked to segment three video clips in the DAVIS 2017 multi-object validation set with satisfactory accuracy as fast as possible, within a 3-min limit. To avoid familiarity bias, they studied the images and ground truths of each video before labeling.

Table 4 reports the Avg(J&F) gain after each interaction. MRIDP_VOS achieves better results within the same number of interactions, which allows our method to converge faster and to a higher final accuracy for experienced users.

## 4. Conclusions

This paper proposes a multi-round interactive bidirectional dynamic propagation instance-level video object segmentation network MRIDP_VOS. A priori segmentation backbone combined high-resolution feature extraction network and convolutional block attention module is proposed to segment objects that the user clicks. In the bidirectional propagation process of segmentation masks, a fusion optimization module was designed to ensure that the user’s interaction intention is not forgotten. Experiments show that compared to the state-of-the-art methods, MRIDP_VOS achieves the best results on various metrics of both interactive segmentation and video segmentation datasets.

## Figures and Tables

**Table 1:** Results of interaction segmentation with different methods. Bold numbers present the best score, and underlined numbers present the second-best score. ‘85%/90%’ denotes NoC@85/90.

Method	Year	SBD		Davis		Berkeley	
		85%	90%	85%	90%	85%	90%
Grabcut [33]	ICCV 01	13.60	15.96	15.13	17.41	12.45	14.22
ESC [34]	CVPR 10	12.21	14.86	15.41	17.70	-	12.11
DOS with GC [35]	CVPR 16	9.22	12.80	9.03	12.58	-	-
LD [36]	CVPR 18	7.41	10.78	5.05	9.57	-	-
f-BRS-B-50 [4]	CVPR 20	4.55	7.45	5.44	7.81	2.17	4.22
FocalClick-18s-S2 [5]	CVPR 22	4.30	6.52	4.92	6.48	1.87	2.86
GPCIS-50 [6]	CVPR 23	**3.80**	**5.71**	4.37	5.89	**1.60**	2.60
PSNet	Ours	4.07	6.18	**3.34**	**4.68**	**1.60**	**1.76**

**Table 2:** Quantitative comparison of different VOS models on DAVIS 2017 validation set. Bold numbers present the best score, and underlined numbers present the second-best score. * denotes results on DAVIS 2016 dataset.

Method	Year	Interactive	*J*↑	*F*↑	Avg(*J*&*F*)↑
IPN [3]	CVPR 19	✓	69.6	73.8	71.7
ATNet [37]	ECCV 20	✓	70.6	76.2	73.4
GNNannot [38]	IJCNN 21	✓	74.8	79.3	77.1
MANet [39]	CVPR 20	✓	76.6	80.7	78.7
MiVOS [7]	CVPR 22	✓	78.9	84.7	81.8
MRIDP_VOS	Ours	✓	**79.8**	**84.9**	**82.4**
XMem [22]	ECCV 22	✗	77.4	84.5	81.0
ISVOS [16]	CVPR 23	✗	79.3	**86.2**	82.8
MED-VT [23] *	CVPR 23	✗	**83.0**	84.1	**83.5**
MRIDP_VOS	Ours	✓	79.8	84.9	82.4

**Table 3:** Quantitative results of ablation study of MRIDP_VOS. Bold numbers present the best score.

	*J*↑	*F*↑	Avg(*J*&*F*) ↑
Standard MRIDP_VOS	**79.8**	**84.9**	**82.4**
(i)	76.6	77.3	77.0
(ii)	77.2	78.9	78.1
(iii)	76.8	78.0	77.4

**Table 4:** Quantitative results of mean incremental Avg(*J*&*F*) after each interaction round. The results in this table are lower than those in Table 2 because the results in Table 2 are the results of more interaction rounds without time constraints.

Methods	Round 1	Round 2	Round 3	Sum
MiVOS	78.6	1.72	0.87	81.2
MRIDP_VOS	79.4	1.83	0.79	82.0

## Data Availability

Data are contained within the article.

## References

[1] Benenson R., Popov S., Ferrari V. Large-scale interactive object segmentation with human annotators. Proceedings of the IEEE/CVF Conference on Computer Vision and Pattern Recognition.

[2] Sofiiuk K., Petrov I.A., Konushin A. Reviving iterative training with mask guidance for interactive segmentation. Proceedings of the IEEE International Conference on Image Processing.

[3] Oh S.W., Lee J.Y., Xu N., Kim S.J. Fast user-guided video object segmentation by interaction-and-propagation networks. Proceedings of the IEEE/CVF Conference on Computer Vision and Pattern Recognition.

[4] Sofiiuk K., Petrov I., Barinova O., Konushin A. f-BRS: Rethinking backpropagating refinement for interactive segmentation. Proceedings of the IEEE/CVF Conference on Computer Vision and Pattern Recognition.

[5] Chen X., Zhao Z.Y., Zhang Y.L., Duan M.N., Qi D.L., Zhao H.H. Focalclick: Towards practical interactive image segmentation. Proceedings of the IEEE/CVF Conference on Computer Vision and Pattern Recognition.

[6] Zhou M.H., Wang H., Zhao Q., Li Y.X., Huang Y.W., Meng D.Y., Zheng Y.F. Interactive Segmentation as Gaussian Process Classification. Proceedings of the IEEE/CVF Conference on Computer Vision and Pattern Recognition.

[7] Cheng H.K., Tai Y.W., Tang C.K. Modular interactive video object segmentation: Interaction-to-mask, propagation and difference-aware fusion. Proceedings of the IEEE/CVF Conference on Computer Vision and Pattern Recognition.

[8] Xu N., Lin W.Y., Lu X.K., Wei Y.C. (2024). Video Object Segmentation: Tasks, Datasets, and Methods.

[9] Caelles S., Maninis K.K., Pont-Tuset J., Leal-Taixé L., Cremers D., Gool V.L. One-shot video object segmentation. Proceedings of the IEEE/CVF Conference on Computer Vision and Pattern Recognition.

[10] Maninis K.K., Caelle S., Chen Y., Pont-Tuset J., Leal-Taixe L., Cremers D., Gool L.V. (2018). Video object segmentation without temporal information. IEEE Trans. Pattern Anal. Mach. Intell..

[11] Khoreva A., Benenson R., Ilg E., Brox T., Schiele B. (2019). Lucid data dreaming for video object segmentation. Int. J. Comput. Vis..

[12] Li X., Loy C.C. Video object segmentation with joint re-identification and attention-aware mask propagation. Proceedings of the European Conference on Computer Vision.

[13] Hu Y.T., Huang J.B., Schwing A.G. Videomatch: Matching based video object segmentation. Proceedings of the European Conference on Computer Vision.

[14] Voigtlaender P., Chai Y., Schroff F., Adam H., Leibe B., Chen L.C. Feelvos: Fast end-to-end embedding learning for video object segmentation. Proceedings of the IEEE/CVF Conference on Computer Vision and Pattern Recognition.

[15] Yang Z., Wei Y., Yang Y. Collaborative video object segmentation by foreground-background integration. Proceedings of the European Conference on Computer Vision.

[16] Wang J.K., Chen D.D., Wu Z.X., Luo C., Tang C.X., Dai X.Y., Zhao Y.C., Xie Y.J., Yuan L., Jiang Y.G. Look Before You Match: Instance Understanding Matters in Video Object Segmentation. Proceedings of the IEEE/CVF Conference on Computer Vision and Pattern Recognition.

[17] Oh S.W., Lee J.Y., Sunkavalli K., Kim S.J. Fast video object segmentation by reference-guided mask propagation. Proceedings of the IEEE/CVF Conference on Computer Vision and Pattern Recognition.

[18] Wang Z., Xu J., Liu L., Zhu F., Shao L. Ranet: Ranking attention network for fast video object segmentation. Proceedings of the IEEE/CVF International Conference on Computer Vision.

[19] Ren S.C., Liu W.X., Liu Y.T., Chen H.X., Han G.Q., He S.F. Reciprocal transformations for unsupervised video object segmentation. Proceedings of the IEEE/CVF Conference on Computer Vision and Pattern Recognition.

[20] Zhou T.F., Wang S.Z., Zhou Y., Yao Y.Z., Li J.W., Shao L. Motion-attentive transition for zero-shot video object segmentation. Proceedings of the AAAI Conference on Artificial Intelligence.

[21] Oh S.W., Lee J.Y., Xu N., Kim S.J. Video object segmentation using space-time memory networks. Proceedings of the IEEE/CVF International Conference on Computer Vision.

[22] Cheng H.K., Schwing A.G. XMem: Long-Term Video Object Segmentation with an Atkinson-Shirin Memory Model. Proceedings of the European Conference on Computer Vision.

[23] Karim R., Zhao H., Wildes R.P., Siam M. MED-VT: Multiscale Encoder-Decoder Video Transformer with Application to Object Segmentation. Proceedings of the IEEE/CVF Conference on Computer Vision and Pattern Recognition.

[24] Dang J.S., Zheng H.C., Wang B.M., Wang L.G., Guo Y.L. (2024). Temporo-Spatial Parallel Sparse Memory Networks for Efficient Video Object Segmentation. IEEE Trans. Intell. Transp. Syst..

[25] Zhang Q., Jin G., Zhu Y., Wei H.J., Chen Q. (2024). BPT-PLR: A balanced partitioning and training framework with pseudo-label relaxed contrastive loss for noisy label learning. Entropy.

[26] Sun K., Xiao B., Liu D., Wang J.D. Deep high-resolution representation learning for human pose estimation. Proceedings of the IEEE/CVF Conference on Computer Vision and Pattern Recognition.

[27] Woo S.Y., Park J.C., Lee J.W., Kweon I.S. CBAM: Convolutional block attention module. Proceedings of the European Conference on Computer Vision.

[28] Hariharan B., Arbel’aez P., Bourdev L., Maji S., Malik J. Semantic contours from inverse detectors. Proceedings of the IEEE International Conference on Computer Vision.

[29] McGuinness K., O’connor N.A. (2010). Comparative evaluation of interactive segmentation algorithms. Pattern Recognit.

[30] Perazzi F., Pont-Tuset J., McWilliams B., Gool L.V., Gross M., Sorkine-Hornung A. A benchmark dataset and evaluation methodology for video object segmentation. Proceedings of the IEEE/CVF Conference on Computer Vision and Pattern Recognition.

[31] Mahadevan S., Voigtlaender P., Leibe B. Iteratively trained interactive segmentation. Proceedings of the British Machine Vision Conference.

[32] Pont-Tuset J., Perazzi F., Caelles S., Arbeláez P., Sorkine-Hornung A., Van Gool L. (2017). The 2017 DAVIS Challenge on Video Object Segmentation. arXiv.

[33] Boykov Y.Y., Jolly M.P. Interactive graph cuts for optimal boundary & region segmentation of objects in ND images. Proceedings of the IEEE International Conference on Computer Vision.

[34] Gulshan V., Rother C., Criminisi A., Blake A., Zisserman A. Geodesic star convexity for interactive image segmentation. Proceedings of the IEEE/CVF Conference on Computer Vision and Pattern Recognition.

[35] Xu N., Price B., Cohen S., Yang J., Huang T. Deep interactive object selection. Proceedings of the IEEE/CVF Conference on Computer Vision and Pattern Recognition.

[36] Li Z., Chen Q., Koltun V. Interactive image segmentation with latent diversity. Proceedings of the IEEE/CVF Conference on Computer Vision and Pattern Recognition.

[37] Heo Y., Jun K.Y., Kim C.S. Interactive video object segmentation using global and local transfer modules. Proceedings of the European Conference on Computer Vision.

[38] Varga V., Lőrincz A. Fast interactive video object segmentation with graph neural networks. Proceedings of the International Joint Conference on Neural Networks.

[39] Miao J., Wei Y., Yang Y. Memory aggregation networks for efficient interactive video object segmentation. Proceedings of the IEEE/CVF Conference on Computer Vision and Pattern Recognition.

